# Exposure to Community Violence and Adverse Childhood Experiences in the Emergency Department

**DOI:** 10.5811/westjem.34857

**Published:** 2025-05-18

**Authors:** Leslie Cachola, Yanina Guevara, Sobia Ansari

**Affiliations:** * Cook County Health, Department of Emergency Medicine, Chicago, Illinois; † Case Western University-University Hospitals, Department of Emergency Medicine, Cleveland, Ohio; ‡ Rush University Medical Center, Department of Emergency Medicine, Chicago, Illinois

## Abstract

**Introduction:**

Adverse childhood experiences (ACEs) and exposure to community violence are public health issues linked to negative mental and physical health outcomes. The emergency department (ED) can play a critical role in the care of patients with a history of trauma exposure. Unfortunately, patients’ experiences often go unidentified, leading to missed opportunities to address and prevent further harm.

**Methods:**

We administered a 22-question survey of trauma exposure in ED patients to 1) identify the prevalence of exposure to community violence and ACEs and resulting post-traumatic stress disorder (PTSD) symptoms, and 2) determine perceived social service needs. This self-administered survey study was conducted on a convenience sample of 267 adult patients at one academic hospital in Chicago, IL, between July 2018–December 2019. This ED sees approximately 70,000 patients annually. These were fluent English-speaking patients who were non-critically ill or altered and chosen randomly after being assigned to an ED room, typically during regular business hours based on research associate availability. They were not offered compensation for study participation. The survey included demographic information and questions modified from the Adverse Childhood Experiences Study questionnaire, the 54-item Survey of Exposure to Community Violence, and the Primary Care PTSD screen. Participants were also asked to identify resources to address their exposure to trauma.

**Results:**

Of 268 surveys, 267 were completed; 88% of participants endorsed exposure to ACEs or community violence (95% confidence interval [CI] 84.1–91.9%, p < 0.001 compared to general US population rate of 61%). A total of 53.6% of respondents endorsed exposure to at least one ACE (95% CI, 47.6–59.6%), and 15.7% were exposed to ≥4 ACE (95% CI, 11.3–20.1%). The most commonly endorsed categories of ACE were “emotional neglect” (30.3%, 95% CI 24.8%–35.8%); “emotional abuse” (25.8%, 95% CI 20.6%–31.1%); and “exposure to family substance use” (21%, 95% CI 16.1%–25.9%). When asked about personal experience with violence in the community, 47.9% said they had been shoved, kicked or punched (95% CI 41.9%–53.9%), 8% had been stabbed (95% CI 4.8%–11.3%), and 6.7% had been shot (95% CI 3.7%–9.7%). Among the survey participants, 26.2% said they had seen someone die from violence either in their home or in their neighborhood (95% CI 20.9%–31.5%). ZIP Code analysis indicates that most patients resided in neighborhoods near our ED and were likely to utilize it for medical care. Of respondents with exposure to trauma 38% asked for resources through their primary care clinic (95% CI 32.2%–43.8%), while 77.4% asked for resources through faith–based organizations (95% CI 72.4%–82.4%).

**Conclusion:**

These findings suggest that most respondents in the ED have experienced trauma, and many are interested in community and clinical resources. These results demonstrate the need for trauma-informed screening in the ED and support for institutional and community-level interventions to address patient experiences.

## INTRODUCTION

Exposure to trauma is a public health issue for adults and children. The emergency department (ED) plays a critical role in caring for patients potentially exposed to trauma. Research has shown that lower socioeconomic status is associated with experiencing and witnessing violence and trauma, particularly for minority communities.^1^–^3^ Patients with lower socioeconomic status have shown a preference for the acute care hospital setting, such as the ED, for medical care compared to ambulatory services due to better access and perceived higher quality of care.^4^ A cross-sectional study of urban ED visits showed a significant increase in pediatric mental health-related ED encounters after exposure to neighborhood gun violence, particularly within two weeks of the shooting and among children living within four to five blocks from the incident.^5^

A study of adults with moderate to severe asthma living in low-income urban neighborhoods demonstrated that violence-exposed participants had 2.27 times more asthma-related ED visits and 2.49 times more asthma-related hospitalizations. Overall, the participants had 1.71 times more ED visits per month.^2^ The ED already plays an important role in the identification and prevention of certain health-related behaviors, such as substance use, tobacco smoking, high-risk sexual practices, and intimate partner violence.^6^–^9^ Zun et al^10^ demonstrated reduced self-reported re-injury rates among victims of interpersonal violence who received interventions in the ED. Generally, however, the ED care experience for patients with a history of trauma is mixed, prompting the need for additional training and implementation of trauma and violence-informed care (TVIC) among emergency clinicians.^11^ Lack of TVIC and adequate patient care plans including TVIC principles may lead to missed opportunities to address and prevent further mental, physical, and emotional harm in susceptible individuals.

We performed a survey of trauma exposure in patients presenting to the Rush University Medical Center (RUMC) ED. An academic medical center located in the Illinois Medical District in Chicago’s near West Side, RUMC includes a 671-bed hospital serving adults and children from the City of Chicago and its eight surrounding counties. The ED delivers care to 70,000 patients per year.

## METHODS

This prospective and self-administered survey study was performed on a convenience sample of patients in the ED of an urban academic medical center between July 2018–December 2019. The survey was only distributed in English. Patients who were approached were chosen randomly after they had been assigned a room in the ED. Patients were offered the survey based on the inclusion and exclusion criteria. They were not offered compensation for study participation. Inclusion criteria for the study were >18 years of age, ability to self-administer the survey, and ability to read and understand English. Exclusion criteria were <18 years of age, inability to self-administer the test, altered mental status, critical illness, and inability to read and understand English.

**Population Health Research Capsule**

What do we already know about this issue?

*Trauma exposure, including adverse childhood experiences (ACEs) and community violence, is a public health issue with significant mental and physical health impacts.*

What was the research question?

*What are the prevalence and effects of trauma exposure in emergency department (ED) patients, and what resources do they need?*

What was the major finding of the study?

*88% of ED patients reported exposure to ACEs or community violence, demonstrating widespread trauma in this population (P<0.0001).*

How does this improve population health?

*Findings highlight the need for trauma-informed care and targeted community resources to address trauma in underserved populations.*

Patients were told that the survey was voluntary and would not affect their care in the ED since research staff were not members of their care team. Patients were informed that the paper survey itself would not contain any identifying information but that the patient’s medical record number, ZIP Code, and chief complaint for that visit would be extracted from their chart. They were offered a written consent form if they agreed to take the survey, which was reviewed and approved by the medical center’s institutional review board. Due to the sensitive nature of the questions, patients were informed of the survey content and that they could discontinue participation at any time.

Study participants were left alone by study staff to complete the survey. Once completed, the surveys were collected by study staff, along with the medical record numbers of study participants to verify the ZIP Code and chief complaint for the ED visit that day. Survey responses were transferred from paper to the password-secured, online platform RedCap (Research Electronic Data Capture hosted at Rush University Medical Center) by the principal investigator (PI) and a study staff member. Responses transferred by the study staff member were reviewed by the PI for accuracy.

### Survey Design

The survey contained 22 questions. The first three questions collected demographic information such as date of birth, race, and sex. The next four questions were borrowed and modified from the Adverse Childhood Experiences Study questionnaire.^12^–^13^ These questions cover childhood abuse, neglect, and household dysfunction. The questions were modified only so that they could be presented in a condensed format in our survey. The next eight questions covered exposure to community violence and were borrowed and modified from the 54-item Survey of Exposure to Community Violence developed by Richters and Saltzman.^14^ These were followed by four questions from the Primary Care Post-Traumatic Stress Syndrome (PC-PTSD) screen.^15^ The final questions asked the study participant to identify the need for resources and specify resources they would find helpful in light of their exposure to trauma.

## RESULTS

**Figure 1:**
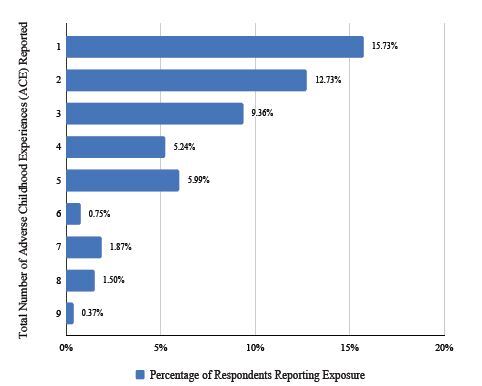
Demographics of 267 survey respondents: 135 (51%) self-identified as Black, 53 (19.8%) Hispanic, 61 (22.8%) non-Hispanic White, 7 (2.6%) Asian, and 9 (3.3%) other. Two survey respondents did not answer this specific question.

A total of 268 surveys were administered, and 267 were completed. As shown in Figure 1, 135 respondents (50.5%) identified as Black, 53 (19.8%) identified as Hispanic, 61 (22.8%) identified as non-Hispanic White, 7 (2.6%) identified as Asian, and 9 (3.3%) identified as other. Of the respondents, 165 (61.8%) identified as female and 96 (36%) identified as male, with one identifying as other. Two respondents did not answer this question specific to demographics.

### Adverse Childhood Experiences

**Figure 2:**
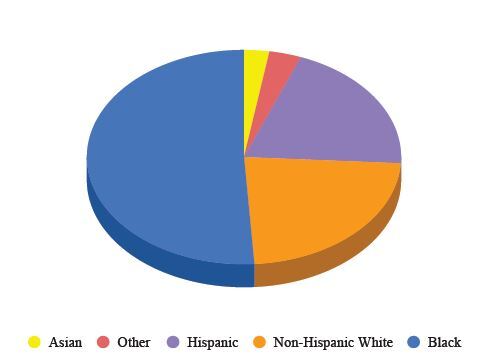
Percentage of survey respondents who reported the number of times they had experienced exposure to adverse childhood experiences (ACEs). Of all 267 respondents, 53.6% experienced at least one ACEs exposure.

As seen in Figure 2, 143 respondents (53.6%) endorsed exposure to *at least* one adverse childhood experience. Forty-two (15.7%) endorsed exposure to one ACE (95% confidence interval [CI] 47.6–59.6%); 34 (12.7%) endorsed exposure to two ACEs; 25 (9.36%) were exposed to three ACEs; 14 (5.2%) were exposed to four ACEs; 16 (5.99%) were exposed to five ACEs; 2 (0.75%) were exposed to six ACEs; 5 (1.87%) were exposed to seven ACEs; 4 (1.5%) were exposed to eight; and 1 (0.37%) was exposed to nine ACEs. 15.7% of total respondents were exposed to 4 or more ACEs (95% CI, 11.3–20.1%). The most commonly-identified ACEs categories were “emotional neglect” (81, 30.3%; 95% CI 24–35.8%), psychological trauma” (69, 25.8%; 95% CI 20.6%–31.1%), and “exposure to family substance use” (56, 21%; 95% CI 16.1%–25.9%).

### Exposure to Community Violence

**Figure 3:**
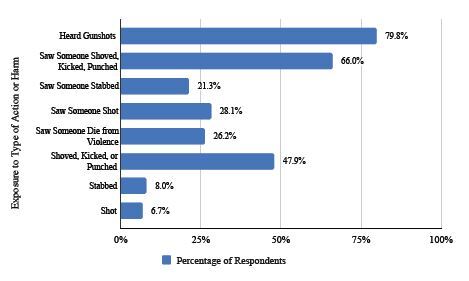
Percentage of respondents’ exposure to community violence based on the type of action or harm.

A total of 213 (79.8%) respondents endorsed hearing gunshots in their communities, which is seen in Figure 3. When asked about witnessing violence in the community, 176 (66%) said they had seen someone shoved, kicked, or punched, 57 (21.3%) had seen someone stabbed, and 28.1% (75) had seen someone shot. 70 (26.2%) of ED patients who responded to our survey said they had seen someone die from violence either in their home or in their neighborhood (95% CI, 20.9%–31.5%). Seventy ED patients who responded to our survey (26.2%) said they had seen someone die from violence either in their home or in their neighborhood (95% CI, 20.9–31.5%). When asked about personal experience with violence in the community, 128 (47.9%) said they had been shoved, kicked, or punched (95% CI, 41.9–53.9%), 22 (8%) had been stabbed (95% CI, 4.8–11.3%), and 18 (6.7%) had been shot (95% CI, 3.7–9.7%).

**Figure 4:**
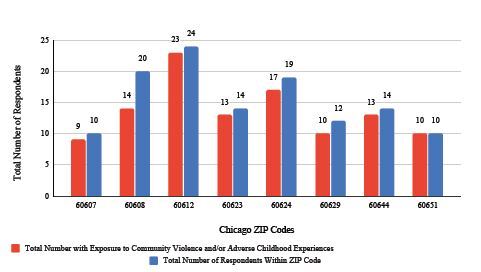
Number of survey respondents with exposure to community violence and/or adverse childhood experiences (ACE) and their respective Chicago ZIP Codes, which are located within the west and south sides of Chicago. The blue bar denotes the total number of patients surveyed from that Zip Code, and the red bar denotes the number of patients who endorsed exposure to community violence and ACEs.

Patients provided their ZIP Codes as part of their demographic information. Figure 4 shows the most frequent ZIP Codes where patients endorsed exposure to violence. The blue bar denotes the total number of patients surveyed from that ZIP Code, and the red bar denotes the number of patients who endorsed exposure to community violence. At least 70% of respondents from all provided ZIP Codes reported exposure to violence.

### Post-Traumatic Stress Disorder Screen

**Figure 5:**
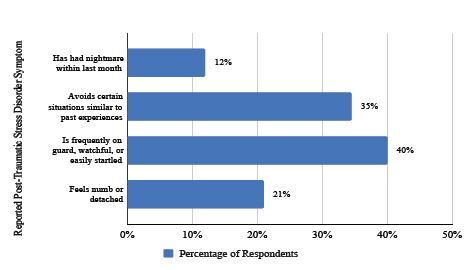
Of 267 respondents, 88% reported exposure to at least one adverse childhood experience or community violence. Of these respondents, 235 (88%) reported post-traumatic stress disorder symptoms as described above.

Patients were asked to answer the PTSD screen based on their responses to the ACEs and community violence questions, a shown in Figure 5. Of 267 respondents, 235 (88%) endorsed exposure to ACEs or community violence (p < 0.001 compared to general US population rate of 61%)^17^. Of these 235 respondents, 28 (12%) endorsed having nightmares in the prior month; 81 (34.5%) endorsed avoiding certain situations that reminded them of their past experiences; 93 (40%) endorsed frequently being on their guard, watchful or easily startled; and 49 (21%) endorsed feeling numb or detached from others, their surroundings or situations.

### Access to Resources

**Figure 6:**
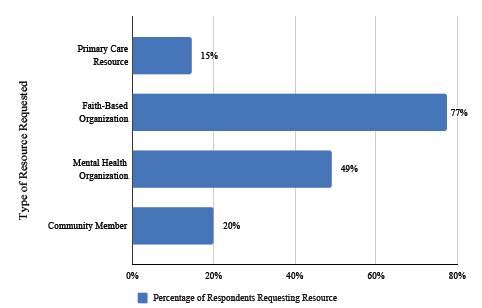
Thirty-eight percent of all respondents with at least one adverse childhood experience requested resources involving primary care clinics (e.g., discussion with primary care physician), faith-based organizations (e.g., discussion with pastor/priest, church attendance, Bible study participation), mental health organizations (e.g., discussion with social worker, counselor), and/or community member support.

The last part of the survey asked respondents whether they would be interested in resources to cope with their experiences and to specify what type of resources in which they would be interested, shown in Figure 6. They were provided some choices and space for free-text responses. Of the 235 survey respondents with exposure to ACEs or community violence, 89 (38%) asked for resources through their primary care clinic (95% CI, 32.2–43.8%). Specifically, 34 (14.5%) said it would be helpful to discuss their concerns with their primary care doctor, and 55 (23.4%) said it would be helpful if their primary care doctor asked them about how they were coping in addition to asking their usual medical questions.

A total of 182 (77.4%) respondents asked for resources through faith-based organizations (95% CI, 72.4–82.4%); 46 (20%) said they would like to speak to a pastor/priest; 53 (22.6%) said they would find it helpful to attend church; 40 (17%) said they would find Bible study class helpful; and 43 (18.3%) said that they would find it helpful to have opportunities to discuss their trauma experiences in a church setting. Of the 115 respondents (49%) who chose mental health organizations as a resource, 69 (29.4%) said it would be helpful to speak with a social worker, therapist, or counselor, and 46 (20%) said it would be helpful to meet with mental health professionals to discuss their trauma experiences. Finally, 46 (20%) respondents said it would be helpful to speak with a community member with similar experiences.

## DISCUSSION

We set out to survey a sample of patients in our ED to better understand ACEs, exposure to community violence, symptoms of PTSD, and the resource needs in our patient population. We created a 22-question survey that included abbreviated forms of the Adverse Childhood Experiences survey, the 54-item Survey of Exposure to Community Violence, and the Primary Care PTSD screening survey.

### Adverse Childhood Experiences

The ACEs section of our survey included the categories of experiences first identified by Felitti and Anda in their seminal study.^12^ The first three categories pertain to personal exposure to abuse, including physical abuse, emotional abuse, and sexual abuse. The next categories pertain to neglect, including physical and emotional neglect. The final categories pertain to household dysfunction such as seeing one’s mother abused or living with someone who used alcohol or illicit drugs, went to prison, or suffered from mental health issues. Similar to the original ACEs study and subsequent others, our study revealed that more than half of respondents had been exposed to ≥1 ACEs. However, 15.7% of respondents endorsed exposure to ≥4 ACEs, much higher than the 6.2% reported in the original study.

This is particularly important given that the study conducted by Felitti noted a graded relationship between number of childhood exposures and adult health risk behaviors and conditions studied: participants with ≥four childhood exposure categories vs none had a 4- to 12-fold increased risk of alcoholism, substance use disorder, depression, and suicide attempts as well as a 2- to 4-fold increased risk of smoking, poor self-rated health, ≥50 sexual partners, and sexually transmitted disease. An increase in the number of categories of ACEs was associated with an increase in the presence of multiple risk factors in adulthood, including ischemic heart disease, cancer, chronic lung disease, fractures, and liver disease. Thus, identifying those with the highest risk of poor health outcomes (eg, exposure to ≥4 ACEs) would allow a more targeted approach to improve care and allot resources for these groups of individuals. The most common ACEs categories identified by our respondents were “emotional neglect” (81, 30.3%); “emotional abuse” (69, 25.8%); and “exposure to family substance use” (56, 21%). The least prevalent category endorsed by our respondents was exposure to sexual abuse (19, 7.1%).

### Community Violence

The next section of our survey included questions divided into witnessing and having personal experiences with violence in the community. Almost 80% of respondents reported that they have heard gunshots in their neighborhoods. More than a quarter had seen someone die from violence, with 21% endorsing seeing someone stabbed and 28% endorsing seeing someone shot in their community. Almost half of respondents endorsed having personal experience with violence in their communities, including being shoved, kicked, or punched.

We examined the ZIP Codes of respondents and found that the highest frequency of ZIP Codes correlated with the greatest exposure to community violence. Most of these ZIP Codes are located on the west side of Chicago and include the communities that we traditionally serve, thus highlighting areas that may benefit from additional medical resources. Notably, Chicago is one of the most segregated cities in the US, and while there have been strides in integration, many neighborhoods remain divided by racial/ethnic and income lines. A large portion of the Black and Hispanic population live on the west and south sides of Chicago, which include University Village (60607), Pilsen (60608), Near West Side (60612), South Lawndale (60623), West Garfield (60624), Chicago Lawn (60629), Austin (60644), and Humboldt Park (60651). These areas have faced disinvestment, crime, and acceleration of health disparities at higher rates.

People from these communities also tend to use the ED more often. According to the Cook County Department of Public Health, South Lawndale (60623) had a 2,797 count of avoidable visits to the ED, while having a primary care physician rate of 75.6 ±7.2% of adults compared to Chicago’s overall rate of 81.1. It stands to reason that if the communities from which the most patients frequent our ED are also experiencing the highest levels of community violence, we have an opportunity to intervene with both medical- and trauma-informed care resources within and outside of the institution.

### Post-Traumatic Stress Disorder

We looked at the responses to the PTSD screen of the respondents with ACEs or exposure to community violence. Many of these respondents were experiencing psychological effects of their exposure at the time of the survey, including feeling guarded or watchful, feeling numb or detached, and avoiding certain triggering situations. These results suggest that many of our patients are experiencing the effects of trauma while also being in vulnerable and challenging situations in the ED. This data should encourage more education and enforcement of trauma-informed care in the ED setting.

### Resources

The majority of respondents with exposure to ACEs or community violence wanted more resources through faith-based organizations. Almost half wanted assistance through mental health organizations and more than a third wanted resources through primary care clinics. More than a fifth of respondents wanted their primary care physician to include questions about coping with trauma exposure in their medical evaluation. These results suggest that while many patients are interested in resources in their communities, there is an opportunity within primary care settings to identify trauma exposure and offer resources.

## LIMITATIONS

There are many limitations to this study. While we interviewed a sample of ED patients, we had to exclude patients who were critically ill or had language barriers, which excluded a significant portion of patients who used the ED. Additionally, we recognize that convenience sampling has an inherent disadvantage, which may result in sampling bias. This was also a survey study, which could have resulted in reporting and recall bias. We acknowledge that this was a small sample size; however, our study came to a halt as COVID-19 measures were put in place and many nonclinical activities were discontinued.

The combination of the three validated surveys has not been validated. While we suspect an association between ACEs, exposure to community violence, and PTSD, an analysis of possible interdependent relationships between these experiences was not the aim of the study. We believe that further research is warranted given that the aforementioned respective studies on each (ACEs, exposure to community violence, PTSD) suggest negative impacts on longitudinal patient health outcomes.

## CONCLUSION

Our survey study revealed that more than half of respondents had been exposed to ≥1 adverse childhood experiences, similar to the original ACE study. However, a far larger proportion was found to have exposure to ≥4 ACEs, while half of all respondents endorsed personal experience with community violence. Of all those exposed to ACEs and community violence, 88% experienced symptoms of PTSD, thus highlighting the importance of trauma-informed care in the ED along with resource allocation and attention toward disproportionately affected communities.

## Supplementary Information
