# Supplementary material for: Exposure to Community Violence and Adverse Childhood Experiences in the Emergency Department
Source: West J Emerg Med. 2025 May 18;26(3):406–12. doi: 10.5811/westjem.34857 (PMC12208022; doi:10.5811/westjem.34857)
Supplement: Supplementary file 1 [file wjem-26-406-s001.docx]

**Appendix 1.** Survey

**Survey #___________**

***Demographics***

1. **Date of birth** _________________________
2. **Race/ethnicity** (choose all that apply):
   1. Black
   2. Hispanic
   3. Asian
   4. non-Hispanic White
   5. Other ______________________
3. **Sex**
   1. Female
   2. Male
   3. Gender-neutral
   4. Other _______________________

***Adverse Childhood Experiences***

While you were growing up, during the first 18 years of your life:

1. Did a parent or other adult in the house often:
   1. Swear at you, insult you, or humiliate you? **Yes / No**
   2. Make you afraid that you may be physically hurt? **Yes / No**
   3. Push, grab, slap, or throw things at you? **Yes / No**
   4. Touch you or have you touch them in a sexual way? **Yes / No**
   5. Try to or actually have oral, anal, or vaginal sex with you? **Yes / No**
2. Did you often feel that:
   1. No one in the family loved you or thought you were important? **Yes / No**
   2. Your family didn’t look out for each other, support each other, or feel close to each other? **Yes / No**
   3. You didn’t have enough to eat, had to wear dirty clothes, and had no one to protect you? **Yes / No**
   4. Your parents were too drunk or high to take care of you or take you to the doctor if you needed it? **Yes / No**
3. Was your mother/guardian:
   1. Often pushed, shoved, grabbed, or had something thrown at her? **Yes / No**
   2. Sometimes or often kicked, bitten, hit with a fist, or hit with something hard? **Yes / No**
   3. Ever repeatedly hit over at least a few minutes or threatened with a gun or knife? **Yes / No**
4. Did you live with anyone who
   1. Was an alcoholic or used street drugs? **Yes / No**
   2. Was depressed, mentally ill, or tried to or did commit suicide? **Yes / No**
   3. Went to prison? **Yes / No**

***Exposure to community violence***

1. Have you ever been shoved, kicked, punched? **Yes / No**
   1. In your home? **Yes / No**
   2. On your street? **Yes / No**
   3. In your neighborhood? **Yes / No**
   4. **Once / More than once** (Circle One)
2. Have you ever seen anyone shoved, kicked, punched? **Yes / No**
   1. In your home? **Yes / No**
   2. On your street? **Yes / No**
   3. In your neighborhood? **Yes / No**
   4. **Once / More than once** (Circle One)
3. Have you ever been stabbed? **Yes / No**
   1. In your home? **Yes / No**
   2. On your street? **Yes / No**
   3. In your neighborhood? **Yes / No**
   4. **Once / More than once** (Circle One)
4. Have you ever seen anyone stabbed? **Yes / No**
   1. In your home? **Yes / No**
   2. On your street? **Yes / No**
   3. In your neighborhood? **Yes / No**
   4. **Once / More than once** (Circle One)
5. Have you ever been shot with a gun? **Yes / No**
   1. In your home? **Yes / No**
   2. On your street? **Yes / No**
   3. In your neighborhood? **Yes / No**
   4. **Once / More than once** (Circle One)
6. Have you ever heard gunshots? **Yes / No**
   1. In your home? **Yes / No**
   2. On your street? **Yes / No**
   3. In your neighborhood? **Yes / No**
   4. **Once / More than once** (Circle One)
7. Have you ever witnessed a shooting? **Yes / No**
   1. In your home? **Yes / No**
   2. On your street? **Yes / No**
   3. In your neighborhood? **Yes / No**
   4. **Once / More than once** (Circle One)
8. Have you ever seen someone die from violence? **Yes / No**
   1. In your home? **Yes / No**
   2. On your street? **Yes / No**
   3. In your neighborhood? **Yes / No**
   4. **Once / More than once** (Circle One)

***Post-Traumatic Stress Disorder Screen***

**If you answered “yes” to any of the previous questions, have those experiences caused you:**

1. To have nightmares in the past month? **Yes or No**
2. To go out of your way to avoid situations that reminded you of those experiences? **Yes or No**
3. To be constantly on guard, watchful, or easily startled? **Yes or No**
4. To feel numb or detached from others, activities, or your surroundings? **Yes or No**

***Category: Resources***

1. If you answered yes to any of the previous questions, would you be interested in resources to help you cope with what you experienced? **Yes/No**
2. What type of resources would help you cope?
   1. Community-based clinic (Aunt Martha’s, Lawndale Christian Center, Miles Square, Access, etc)
      1. Would it be helpful to discuss your concerns with your primary care doctor? **Yes / No**
      2. When you meet with your doctor would you find it helpful if he/she asked about your coping concerns as well as your medical concerns? **Yes / No**
   2. Faith-based organization
      1. Would it be helpful to speak with your pastor/priest? **Yes / No**
      2. Would it be helpful to attend church? **Yes / No**
      3. Would it be helpful to go to a Bible study class? **Yes / No**
      4. Would you find it helpful if there was an opportunity at church to discuss traumatic experiences or coping concerns? **Yes / No**
   3. Mental health clinic
      1. Would it be helpful to talk to a counselor, social worker, or therapist (C4, Thresholds, etc) **Yes / No**
      2. Would you find it helpful if you could meet with a mental health professional to talk about your coping concerns? **Yes / No**
   4. Peer-based organization (AA/NA, mentorship programs)
      1. Would you find it helpful if you could talk to people in your community who have experienced similar things? **Yes / No**
3. What other resources do you think would help you?

*AA*, Alcoholics Anonymous; *NA*, Narcotics Anonymous.________________________________________________________________

________________________________________________________________
